# Feasibility Study on the Classification of Persimmon Trees’ Components Based on Hyperspectral LiDAR

**DOI:** 10.3390/s23063286

**Published:** 2023-03-20

**Authors:** Hui Shao, Fuyu Wang, Wei Li, Peilun Hu, Long Sun, Chong Xu, Changhui Jiang, Yuwei Chen

**Affiliations:** 1 School of Electronics and Information Engineering, Anhui Jianzhu University, Hefei 230601, China; 2 Anhui International Joint Research Center for Ancient Architecture Intellisencing and Multi-Dimensional Modeling, Hefei 230601, China; 3 Institute of Unmanned System, Beihang University, Beijing 100191, China; 4 Department of Remote Sensing and Photogrammetry, Finnish Geospatial Research Institute, 02150 Espoo, Finland; 5 Ji Hua Laboratory, Foshan 528200, China

**Keywords:** hyperspectral LiDAR, spectral feature, classification, edge point, reprogramming

## Abstract

Intelligent management of trees is essential for precise production management in orchards. Extracting components’ information from individual fruit trees is critical for analyzing and understanding their general growth. This study proposes a method to classify persimmon tree components based on hyperspectral LiDAR data. We extracted nine spectral feature parameters from the colorful point cloud data and performed preliminary classification using random forest, support vector machine, and backpropagation neural network methods. However, the misclassification of edge points with spectral information reduced the accuracy of the classification. To address this, we introduced a reprogramming strategy by fusing spatial constraints with spectral information, which increased the overall classification accuracy by 6.55%. We completed a 3D reconstruction of classification results in spatial coordinates. The proposed method is sensitive to edge points and shows excellent performance for classifying persimmon tree components.

## 1. Introduction

The persimmon, cultivated in China for more than 3000 years, is a nutritious fruit containing a large amount of sugar and various vitamins. The development of persimmon farming has resulted in numerous orchards with high automation management, such as fertilization, automatic fruit picking or package, and irrigation. In order to manage persimmon trees precisely and intelligently, it is essential to have abundant and readily available information that can record their growth stages based on the structural components, including leaves, fruit, and wood. However, it is difficult to accurately measure and describe these components due to their spatial variability and structural complexity of the persimmon tree. Therefore, there is an urgent need for a method to separate and classify persimmon trees’ components.

To detect and classify fruit trees’ components, researchers utilize different methods, including visible cameras, multispectral/hyperspectral cameras, LiDAR, and these methods in combination. Several methods have been developed to obtain information from trees under various natural conditions with visible light image processing, such as fruit target detection [1,2], segmentation of green object fruit under complex orchard backgrounds [3], segregation of tomato phenotypes [4], and fast extraction of tree canopy areas from UAV images [5].

The evolution of hyperspectral imaging technology has driven a more comprehensive extraction of fruit tree data. Varga et al. established an evaluation model of hyperspectral images combined with deep neural networks to discriminate and visualize different fruit ripeness levels [6]. The discrepancy in reflectance at specific wavelengths was used to detect potentially damaged fruit [7,8]. The capabilities of hyperspectral imaging have also been explored for applications such as fruit identification and detection [9], nondestructive testing of dry matter [10], moisture content estimation [11], etc. However, the natural defects of images and passive spectral information may be disturbed by factors such as illustration condition, shadows, occlusion, complex branch structures and understory layers, etc., which will inevitably lead to a reduction in wood, leaf, and fruit recognition accuracy in the understory [12].

Time-of-flight measurement is used in pulsed LiDAR for range measurement. Thus, LiDAR can obtain an accurate and instant distance image of the target, which drives processing and learning for autonomous driving [13], while in precision agriculture and smart forestry, LiDAR are used to monitor and reconstruct 3D models of fruit tree components [14]. In existing studies, researchers have employed LiDAR to extract tree height [15], tree branch topology [16], and fruit location [17]. LiDAR can measure distance accurately and obtain spatial information of fruit trees efficiently, but the laser’s monochromatic nature limits its ability to provide abundant spectral information.

Combining several monochromatic laser sources or fusing LiDAR and multispectral/hyperspectral data is an instant method used to obtain fine spatial-spectral information. In a study, researchers fused different wavelengths of LiDAR to complete tree wood and leaf component separation [18,19,20]. This general fusion strategy to meet quantitative analysis requirements has only four to eight channels of spectra, and the spectral resolution is insufficient [21]. Another option is fusing LiDAR and hyperspectral data to generate spatial-spectral domain data for describing tree composition [22]. However, combining too many laser sources is problematic in extending spectral band coverage and improving spectral resolution, resulting in higher hardware costs and more complicated registration. The latest developed active remote sensing system, hyperspectral LiDAR (HSL), can obtain spatial and spectral information simultaneously without any external illumination [23]. Nevalainen et al. searched for two vegetation indices sensitive to nitrogen concentration and verified the possibility of the 3D estimation of nitrogen with HSL data [24]. Bi et al. established a partial least squares regression model to achieve the inversion of chlorophyll concentration at any vertical position in maize plants with HSL spectral and spatial information [25]. To explore the potential of HSL applications in forestry development, Hakala et al. presented the first scheme for modeling and assessing the three-dimensional distribution of chlorophyll concentration and water content in Norway spruce based on a full-waveform HSL [26]. After that, Vauhkonen et al. explored HSL’s feasibility for tree species classification using similar techniques [27]. For the study of tree components’ classification, most previous studies have focused on wood-leaf classification [28] and neglected undesirable fruit extraction. Therefore, the simultaneous monitoring of fruit, leaves, and wood in precision agriculture and forestry has become an urgent problem to be solved.

This paper explores the feasibility of the classification of persimmon trees’ components under laboratory conditions with a revised 101-channel HSL system. We proposed a classification method with spectral and spatial characteristic parameters to classify four components and indicate classification results with 3D reconstruction. Firstly, we extracted nine characteristic parameters in the spectral domain based on persimmon tree HSL point cloud data. Then, a preliminary classification of the fruit trees’ components was conducted with characteristic spectral parameters. To solve the misclassification of edge points, we propose an enhanced classification method for edge-points-based spatial constraint relationships among point clouds. Finally, the results of tree component classification were fused into spatial coordinates to accomplish 3D reconstruction of a persimmon tree.

## 2. Materials and Methods

### 2.1. Hyperspectral LiDAR System

**Figure 1:**
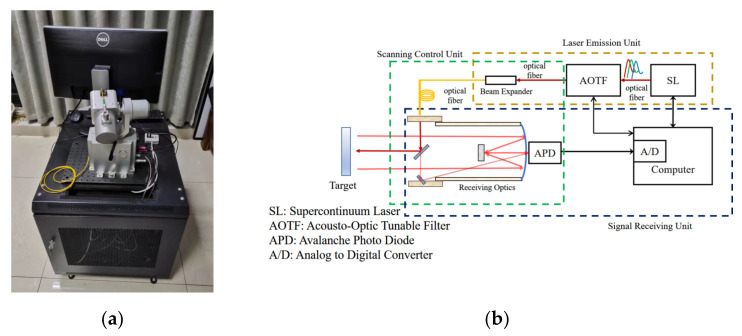
Schematic of the HSL system: (**a**) installation and (**b**) system schematic; The red arrow represents the optical signal, while the black arrow represents the electrical signal.

Figure 1 shows the structure of a hyperspectral LiDAR system which consists of an emission unit, an integrated scanning control unit, and a receiving unit. The emission unit’s acoustic-optically tunable filter (AOTF) provides a detection laser with a spectral resolution of 5 nm from 550 nm to 1050 nm by filtering the outgoing laser from the super continuous laser. The HSL system emits a 10 mm diameter laser beam with a divergence angle of 1 mrad, and the transmitted laser beam is collimated by a collimator with a focal length of 33 mm, resulting in a 5–8.5 mm spot diameter of the emitted laser after collimation. A two-axis rotator of a scanning control unit conducts precise scanning to generate final colorful point cloud of a target. The laser echoes reflected from the target are focused on an avalanche photon diode (APD) by the receiving optics, which are captured and stored by a high-speed data acquisition card (AC) for subsequent processing.

### 2.2. Experimental Samples

**Figure 2:**
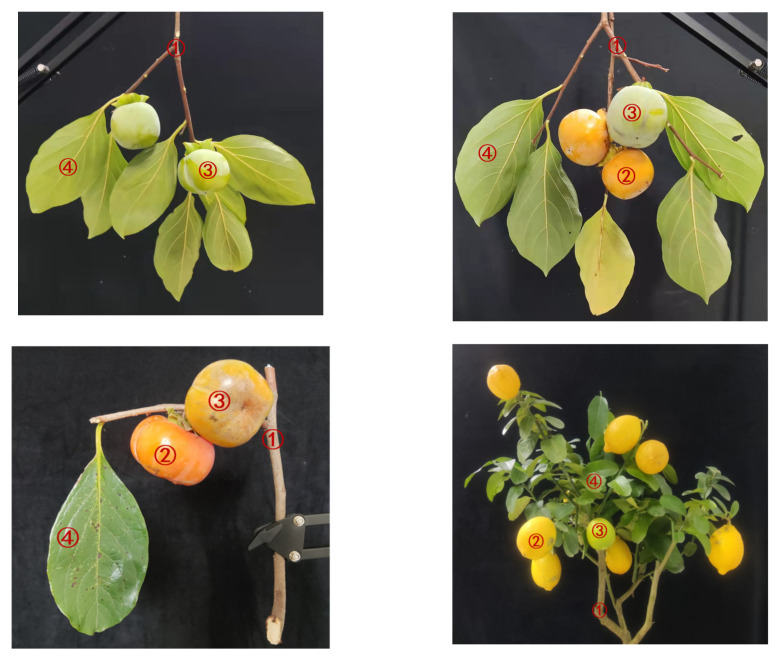
Fruit tree samples. ①, ②, ③, and ④ correspond to the components of the wood, ripe fruit, unripe fruit, and leaves, respectively.

To evaluate and verify the performance of the classification method in the next section, we acquired spatial–spectral point cloud data of tree samples from our HSL. The tree samples include two species: persimmon (*Diospyros kaki Thunb.*) and lemon (*Citrus limon (L.) Burm. F.*). The persimmon samples we used for the study included six branches with unripe fruit and six branches with both ripe and unripe fruit. All fresh branches were sawn off from persimmon trees in the South Campus of Anhui JianZhu University in October 2021 and July, August, and September 2022. In addition, we selected three lemon trees (bonsai trees), which were used to simulate tree samples for an orchard, to explore the generalizability of our method to other fruit tree species. For persimmon samples, the spectral characteristics of unripe and mid-ripe fruits are similar, while the spectral characteristics of ripe and over-ripe fruits are similar [29]. Therefore, this study defines two ripeness levels (ripe and unripe) to characterize fruit ripeness. The lemon samples were from trees at the mid-ripening stage with unripe and ripe fruit. As illustrated in Figure 2, the order numbers ①, ②, ③, and ④ correspond to the components of the wood, ripe fruit, unripe fruit, and leaves, respectively. All samples were hung vertically on a metal stand at 10 cm from the black cloth behind the sample.

### 2.3. Data Acquisition and Processing

**Figure 3:**
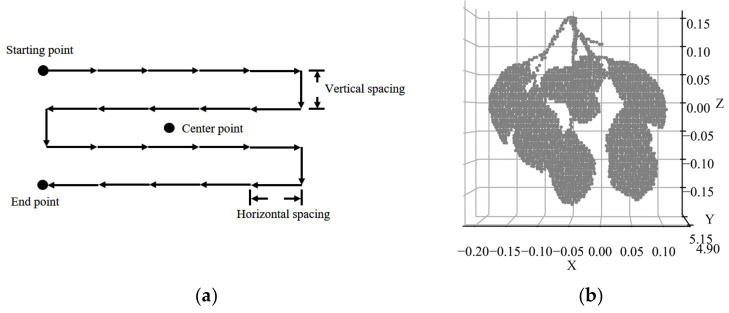
HSL scanning strategy and persimmon tree scan points. (**a**) The zigzag scanning pattern of HSL; (**b**) preprocessed HSL point cloud of the persimmon tree.

Data acquisition was conducted in a laboratory environment where the samples were placed at a horizontal distance of 5 m in front of the HSL system. We made the point cloud cover the whole sample by setting appropriate pitch and horizontal steps for the scanning unit. Figure 3a shows the zigzag scanning pattern, which starts at the start point (the top left corner of the target). Scanning is accomplished at the endpoint following the direction of the arrow, and the vertical and horizontal scanning step are both 0.05 radians for generating a dense but evenly distributed point cloud. The HSL point cloud includes spatial coordinates and full waveform signals of 101 wavelengths that are recorded and stored in real time by a two-axis rotator actuator.

Prior to data collection, a standard 99% reflectivity diffuse reflection whiteboard (TD-MEB99-141Y-20) as a reference whiteboard in front of a black fabric with less stray light was scanned using the HSL system. Samples placed at the same distance as the whiteboard were scanned immediately. The intensity values of the sample and reference whiteboard were used to calculate the reflectance of the sample [27], as shown in Equation (1). Here, $\rho_{t}\left(\lambda_{i}\right)$ is the reflectance value of each wavelength of the sample, *I* indicates the 101 spectral channels of the HSL system, $V_{t}\left(\lambda_{i}\right)$ and $V_{b}\left(\lambda_{i}\right)$ are the peak voltage of the HSL echo signal at wavelengths for the sample and the reference whiteboard, respectively, and $\rho_{b}\left(\lambda_{i}\right)$ is the reflectivity value of the reference whiteboard.

(1) $$\rho_{t}\left(\lambda_{i}\right)=\frac{V_{t}\left(\lambda_{i}\right)}{V_{b}\left(\lambda_{i}\right)}\cdot \rho_{b}\left(\lambda_{i}\right)$$

To eliminate the interference of the background echo signal, we performed point cloud segmentation. First, we used the difference in spatial coordinates between the background cloth point cloud and the sample point cloud on the Y-axis to separate the sample and the background with a fixed distance value. The complete sample scan points were obtained (Figure 3b).

## 3. Methods

**Figure 4:**
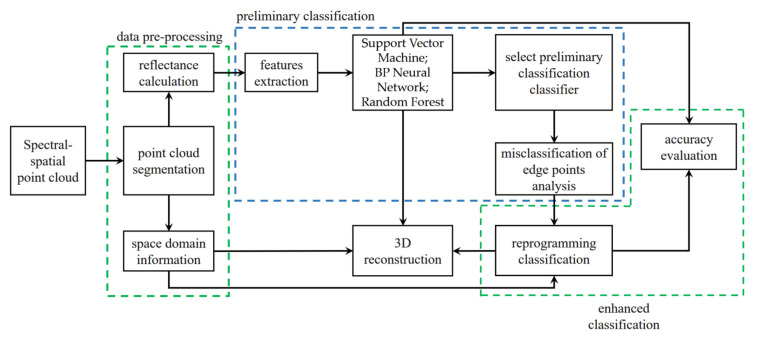
Structure diagram of tree components’ classification and 3D reconstruction.

Figure 4 shows a schematic diagram of the proposed classification method of persimmon trees’ components, which includes four parts: data preprocessing, preliminary classification, enhanced classification, and 3D reconstruction.

The data preprocessing part consists of point cloud segmentation and spectral reflectance calculation, which was discussed in Section 2.3. The preliminary classification includes feature parameter extraction (refer to the analysis in Section 3.1 for details), multiple classifications, and misclassification of edge points analysis. In order to reduce the misclassification of edge points, enhanced classification was conducted based on the spatial constraint relationship of the HSL point cloud. Finally, the 3D reconstruction of the classification results was completed by fusing the spatial coordinates.

### 3.1. Feature Parameter Extraction

**Figure 5:**
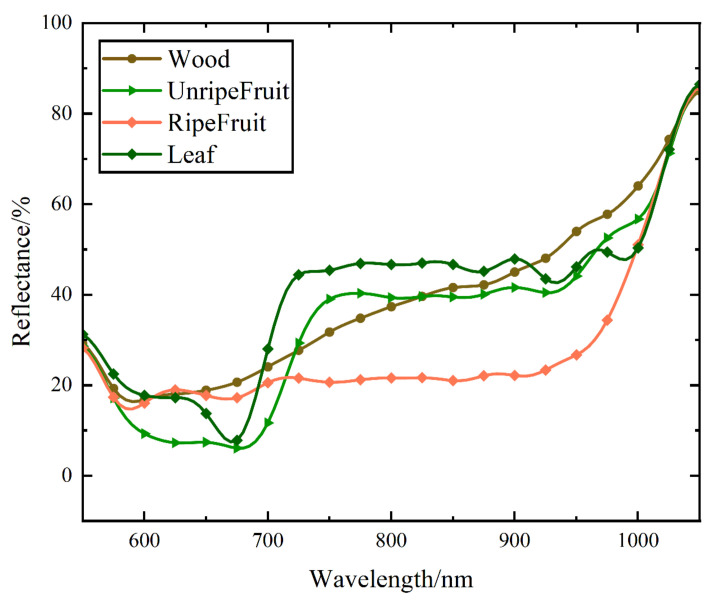
The reflectance of persimmon tree components.

In order to analyze the reflection properties of the persimmon tree samples across different wavelengths, we plotted the average reflectance distribution curves for each of the four components in Figure 5. The reflectance variation tendencies of the four components are considerably different, as observed. The reflectance of the wood increases with the wavelength. The leaf reflectance has a clear red-edge effect, with low reflectance in visible bands and high reflectance in near-infrared bands [30]. In addition, the reflectance of unripe fruit is similar to the leaves, with a clear red-edge effect, indicating that unripe fruit contains a certain amount of chlorophyll. The ripe fruit reflectance distribution tends to stabilize at 20% in the spectral range from 600 to 900 nm without an obvious red-edge effect.

Feature parameter selection should be based on practical application and classification performance considerations. We selected feature parameters based on the differences in the spectra of the four components, which can effectively retain the physical information of persimmon spectra while avoiding the information loss and computational complexity issues that may arise from traditional dimensionality reduction algorithms [31]. Selecting vegetation indices as parameters can also eliminate errors caused by laser incidence angles [32]. Considering the difference in the components’ spectral reflectance, we selected five reflectance values as the feature parameters with the largest differences at typical bands (700 nm, 730 nm, 780 nm, 850 nm, and 900 nm), defined as R_700_, R_730_, R_780_, R_850_, and R_900_, respectively, based on the maximization of interclass variance. The R_700_ and R_730_ bands, which are sensitive to chlorophyll, accentuate the contrast between components with chlorophyll and those lacking chlorophyll. R_780_ is the reflectance of the band with the largest difference in reflectance between the four components. The reason for choosing R_850_ and R_900_ was to reflect the difference in reflectance between wood and other persimmon components. The average value (AVG R_760_–R_930_) of the reflectance in the range (760 nm–930 nm) with the large spectral differences between these four components was used as a feature parameter. The red-edge chlorophyll index (CI _red edge_) [33] was selected as a feature parameter based on the red-edge effect due to chlorophyll’s absorption of visible light. The normalized difference vegetation index (NDVI) [34] and the normalized difference red-edge index (NDRE) [32] were selected to distinguish the wood, leaf, and fruit components. The specific parameters are listed in Table 1.

### 3.2. Preliminary Classification

We focused on the random forest (RF), support vector machine (SVM), and BP neural network (BPNN) methods, which are machine learning methods that have demonstrated excellent classification performance in previous remote sensing studies [35,36,37]. After nine feature parameters in the spectral domain were chosen, we investigated the performance of the SVM, BPNN, and RF methods in the classification of persimmon trees’ components; we selected the best one as a preliminary classifier.

Binary decision trees are used as the basic building blocks of RF [38], combined with the basic building blocks for training and prediction to achieve the resultant output of each decision tree, and, finally, they use voting for the plurality to obtain the final classification result. Eight decision trees were selected to build the classification trees, and four classes (wood, leaf, ripe fruit, and unripe fruit) were classified by sampling the data with replacement.

The central idea of the SVM classification algorithm is to maximize the optimal hyperplane as the decision function. The hyperplane used to separate the data is also known as a support vector, and the optimal hyperplane is created by accurately separating each data point and ensuring that the distance between classes is maximized [39]. We used spectral features as the input to the SVM and four persimmon tree component labels as the output vectors, and a common radial basis function was selected as the kernel function. SVM parameters, including the kernel function and penalty parameters, were selected with default values of 0.1 and 10, respectively.

The core idea of the BPNN method is to reasonably distribute all features into a uniform feature space. The BPNN method accomplishes the corresponding clustering or classification of data by constructing nonlinear functions and optimizing the loss function to fit data cells in the target domain [40]. In this paper, we constructed a total of nine input neurons and one hidden layer, and the number of hidden layer nodes was five according to the empirical equation; the active function was the sigmoid function, and, finally, the number of output neurons was four.

First, we selected six persimmon tree samples and three lemon tree samples as the training samples for preliminary classification. We manually labeled the training point clouds by compared with RGB images in CloudCompare (version 2.11.3, CloudCompare SAS, F-34000, Montpellier, France). The labeled data were extracted from training point cloud data and we completed preprocessing. Then, the labeled data were randomly divided into two sets in the ratio of 7:3, i.e., a training set and a validation set. Finally, we input the selected parameters of each point cloud as the training data.

### 3.3. Enhanced Classification

**Figure 6:**
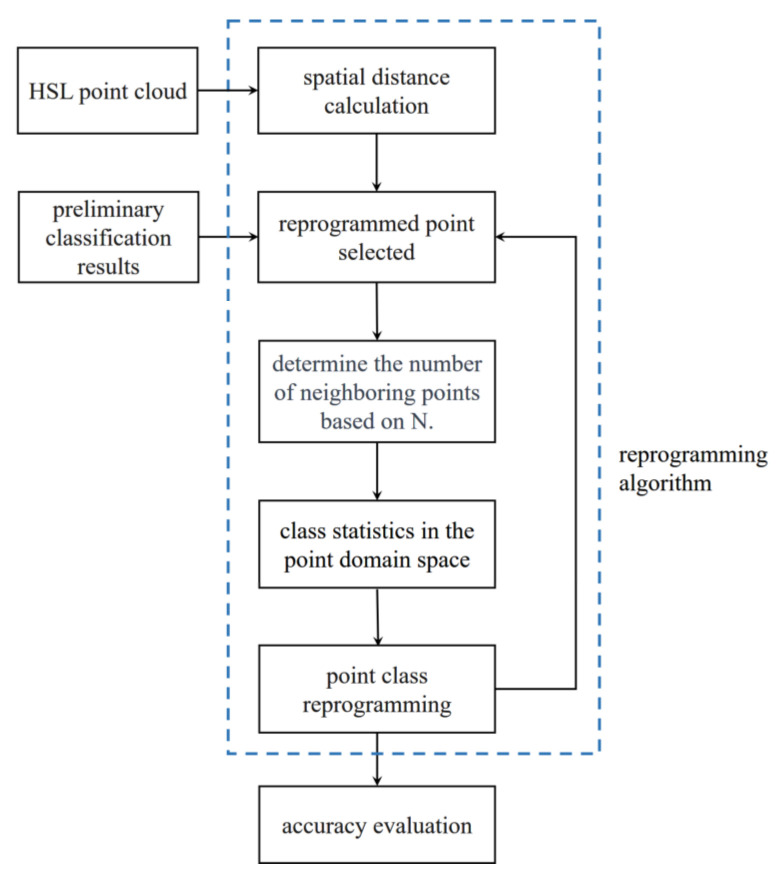
Enhanced reprogramming algorithm based on spatial distance.

We used only spectral domain feature parameters in the preliminary classification, causing the misclassification of edge points (we will analyze the reasons in detail in Section 4.1). To enhance the accuracy of the classification, especially correcting the misclassification points at the edges, we proposed an enhanced classification method based on spatial distance, which reprograms the edge point class, according to the class consistency of adjacent spatial points [41]; its block diagram is shown in Figure 6.

First, spatial distances of all points were calculated to generate an initial distance matrix, which, together with the preliminary classification results, was used as the input to the reprogramming algorithm. The initialized distance matrix *S* was calculated as Equation (2) by the Euclidean distance between point i and point j in the sample point cloud.

(2) $$S(i,j)=\sqrt{{\left(i_{x}-j_{x}\right)}^{2}+{\left(i_{y}-j_{y}\right)}^{2}+{\left(i_{z}-j_{z}\right)}^{2}}$$

The reprogramming algorithm consists of four steps, as shown in the blue box in Figure 6.

Step 1—reprogrammed point selected: Take a sample point *k* that belongs to a class decided by the preliminary classification results and sort the distance S(k,j) from *k* to any other sample point j.

Step 2—adjacent points decision: Establish a matrix *S_N_*, which includes the *N* smallest distance points in the point *k* neighborhood, where *N* is the empirical value obtained from our multiple experiments.

Step 3—class statistics in the point domain space: Count the number of four classes separately in *S_N_*, select the largest proportion of class labels as the point *k* label, and complete the class rewriting of point *k*.

Step 4—point class reprogramming: After the *k*-point label has been rewritten, return to step 1 and iterate through all of the sample points in the sequence until all of the points are reprogrammed.

### 3.4. Three-Dimensional Reconstruction

The target point cloud includes spatial-spectral information, which provides the basis for 3D reconstruction after classifying persimmon tree components. In this study, we used color mapping to display the point cloud classification results, providing an intuitive visualization of the spatial distribution of the different classes. By assigning unique color to each class, we could differentiate and identify each point based on its classification. Three-dimensional reconstruction of different sample trees’ components was accomplished in the Python 3.6 environments.

### 3.5. Accuracy Evaluation

In previous point cloud classification studies, the correctness of validation sets has often been used to evaluate the performance of classification algorithms. However, generalization errors often exist due to certain factors, such as insufficient data in the validation set and overfitting of the model during classification. To evaluate the performance of our classification method in the HSL point cloud, we proposed an accuracy evaluation method. First, we manually annotated the sample point cloud in CloudCompare to create a real dataset with spatial coordinate information and labeled values.

The real label dataset *Q* is built as Equation (3).

(3) $$Q=\left\{q_{i}|i\in \left[1,n\right]\right\},q_{i}=\left\{X_{qi},Y_{qi},Z_{qi},{label}_{qj}\right\}$$

n is the overall number of points in the sample point cloud, and $X_{qi},Y_{qi},Z_{qi}$ are the 3D coordinates of point i. ${label}_{qj}$ denotes class labels, *j* ranges from 1 to 4, and their corresponding classes are {UnripeFruit,RipeFruit,Wood,Leaf}.

Similarly, we can build the predicted label dataset P, as shown in Equation (4).

(4) $$P=\left\{p_{i}|i\in \left[1,n\right]\right\},p_{i}=\left\{X_{pi},Y_{pi},Z_{pi},{label}_{pj}\right\}$$

Then, we calculate the correct prediction points. We set the number of correct classified points for each class in the prediction set P as $T_{j0}$ and the number of correct classified points for each class in the true set Q as $H_{j0}$. The initial values $H_{j0}$ and $T_{j0}$ are both 0. We loop through the prediction set and the true set and compare their label values at the same coordinate position, i.e., $X_{pi}=X_{qi},Y_{pi}=Y_{qi},Z_{pi}=Z_{qi}$ is satisfied. If ${label}_{pj}={label}_{qj}$ is satisfied, $T_{j0}=T_{j0}+1,H_{j0}=T_{j0}+1$, otherwise $T_{j1}=T_{j0},H_{j1}=T_{j0}+1$. When all of the points in P and Q are compared, we can obtain the final $T_{jn}$ and $H_{jn}$.

Finally, the classification accuracy of one class is defined as Equation (5).

(5) $$K_{j}=\frac{T_{jn}}{H_{jn}}$$

The overall classification accuracy ($K_{Overall}$) is determined as Equation (6), which is the quotient that divides all of the correctly classified points in the prediction set by all of the sample points.

(6) $$K_{Overall}=\frac{\sum_{i=1}^{j}T_{i}}{\sum_{i=1}^{j}H_{i}}$$

## 4. Results and Discussion

### 4.1. Preliminary Classification Performance

**Figure 7:**
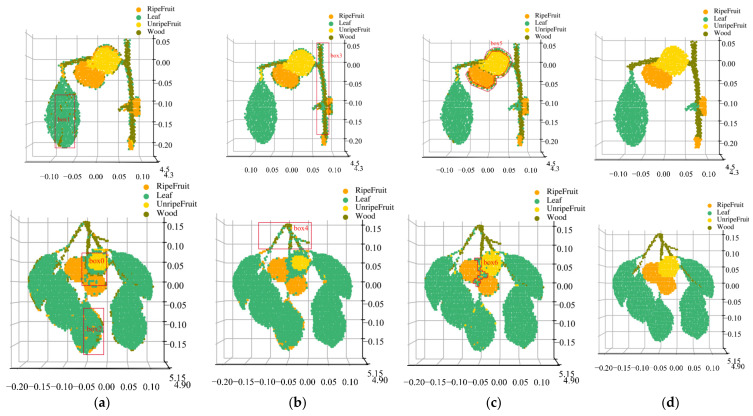
Three-dimensional reconstruction of the preliminary classification of the persimmon sample. (**a**) Support vector machine classifier; (**b**) backpropagation neural network classifier; (**c**) random forest classifier; (**d**) real class labels of the persimmon samples.

Figure 7 illustrates the reconstructed results of three classifiers for some of the persimmon samples (Figure 2). As we can observe, the SVM method has the lowest accuracy, as it cannot classify the corresponding target class at the edge correctly, and it also presents misclassification at some leaf and fruit nonedge areas, such as red boxes 0, 1, and 2. The reconstructed image with the BPNN method can correctly classify most of the sample points, although there are some misclassified points at the edges of the wood and the fruit edges, as shown in boxes 3 and 4. Finally, the RF classifier has the highest accuracy as four classes of the persimmon trees’ components can be distinguished, but there are some misclassified points at the edges of the fruit, as shown in boxes 5 and 6.

Table 2 lists the classification accuracy of the nine spectral domain parameters by the SVM, BPNN, and RF classifiers. All three classifiers demonstrate excellent classification performance, with the RF classifier exhibiting the highest accuracy and the SVM classifier showing the lowest accuracy. The three algorithms’ overall accuracy values are 84.6%, 86.3%, and 88.6%, respectively. The mean accuracy of the leaf is greater than 87%. The maximum accuracy of the ripe fruit is 85.5% with the RF classifier, the maximum accuracy of the unripe fruit reaches 86.2% with the BPNN classifier, and the accuracy of the wood is below 82% with all three classifiers. We selected the best one, RF, as the preliminary classification classifier on all these counts.

To analyze misclassification with the RF method at edge points, we manually extracted the edge and nonedge region points of the four components of the persimmon tree samples (Figure 7c), selected more than half of the points, and calculated the average spectral reflectance.

**Figure 8:**
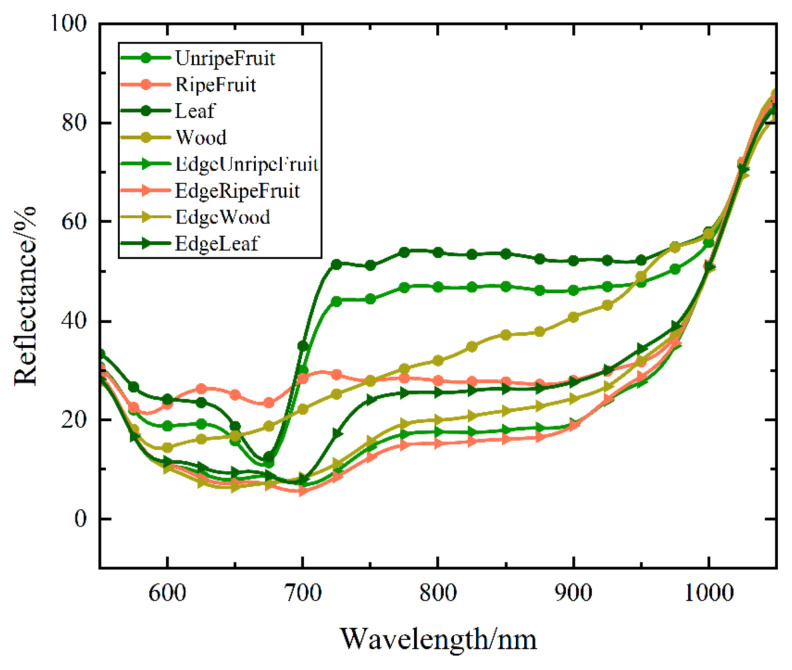
Reflectance of the edge and nonedge of the persimmon components.

As shown in Figure 8, there are obvious reflectance differences between the edge and nonedge points. The nonedge reflectance of unripe fruit is 10.36% higher than its edge average, and there is a clear red-edge effect on the spectral reflectance of nonedge points, while the spectral reflectance of edge points shows a slowly increasing trend. The nonedge point reflectance of ripe fruit is 17.81% higher than that of the edge point on average, and the spectral reflectance is more flatly distributed in the range of 600 nm–900 nm, while the spectral reflectance at the edge shows a slowly increasing trend. The nonedge reflectance of the leaf and wood are, on average, 18.18% and 11.06% higher than their corresponding edge points, respectively, and their edge reflectance curves have a similar trend. The overall reflectance curve trends similarly at the edge of the four component classes, while the leaf edge spectral reflectance is higher. In summary, the spectral differences between the edge points and nonedge are the main reason for the misclassification of the edge points with reflectance as a feature for classification only.

In the preliminary classification, the parameters were selected only from the spectral data, which were calculated based on the peak value of the LiDAR echo signals [28]. However, when the HSL system collects data at fruit or wood edges, the calculated reflectance often differs from the nonedge’s (the large incidence angle at the fruit’s edges can also lead to abnormal reflectance). The reason is that part of the HSL signal spot falls on the background, covers multiple components, or even misses the target, causing error in the collected echo signal [42].

### 4.2. Enhanced Classification Performance

#### 4.2.1. Neighboring Point Decision

Table 3 lists the enhanced classification average accuracy of persimmon tree samples with different numbers of neighboring points (N). The proposed method can obtain the highest classification accuracy when N is 12, so we selected 12 as the N value in the following sections.

#### 4.2.2. Classification Performance

The accuracy of the persimmon components with preliminary classification versus our enhanced classification is listed in Table 4. The overall accuracy of our classification method increases to 96.6%, an 8% gain over the preliminary classification. Through the spatial features, the gains of our classification method over preliminary classification are 12.9%, 12.4%, 12.2%, and 2.3% on the unripe fruit, ripe fruit, wood, and leaf, respectively. Therefore, the proposed method outperforms classification that uses spectral features in each component’s experimental conditions, which benefits from the fact that our method can preserve the spatial structure and reveal the constrained dependencies between points besides the spectral features.

The classification results of the lemon trees by different methods is listed in Table 5. The overall accuracy values of the SVM, BPNN, and RF methods in the preliminary classification are 80.1%, 84.9%, and 88.3%, respectively. The BPNN classification accuracy values of the wood and unripe fruit are 78.8% and 88.4%, respectively. The RF method has the highest classification accuracy for the leaf and ripe fruit, with 89.3% and 83.3%, respectively, and the SVM classifier has the worst classification accuracy. Following the enhanced classification of the lemon sample, the accuracy of our method increased by 5.1% over the preliminary classification and could reach 93.4%. Compared to the preliminary classification using spectral features, the classification accuracy values of the leaf, ripe fruit, unripe fruit, and wood with our method increased by 5.4%, 9%, 3.7%, and 17.4%, respectively. The overall accuracy of the lemon sample was lower than that of the persimmon sample as the lemon tree had a more complex spatial structure.

### 4.3. Reconstruction of the Classification Results

**Figure 9:**
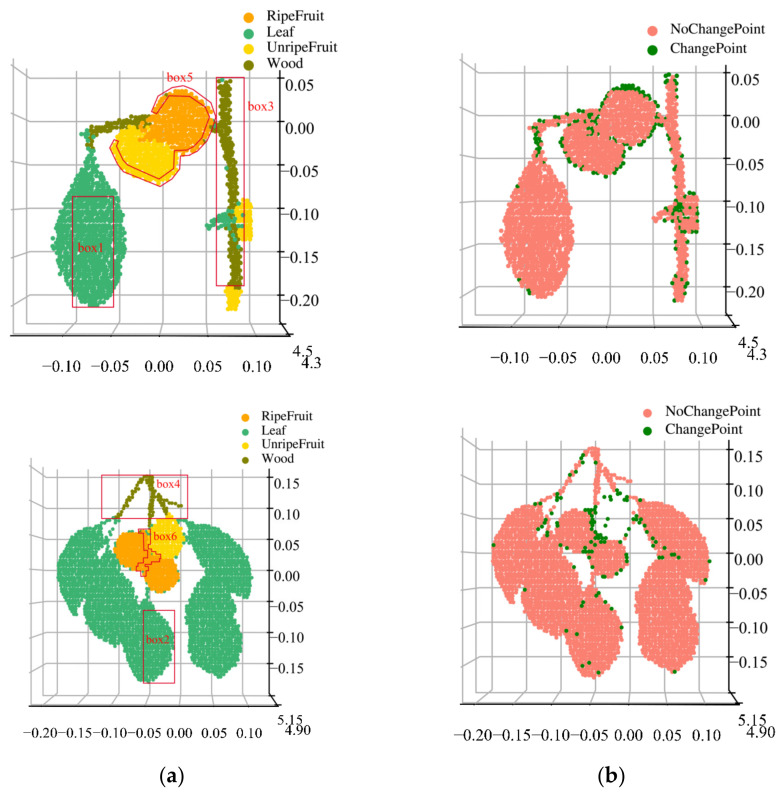
Reconstruction of classification results and changes in point cloud class. (**a**) Reconstruction based on the proposed method and (**b**) class changes in the reprogramming strategy. The red boxes are the areas misclassified in the preliminary classification.

Figure 9a shows the classification result reconstruction of the two persimmon samples. Our method can effectively distinguish different tree components; in particular, the point class in the edge areas can still be corrected during preliminary misclassification. Compared to Figure 7, the leaf and wood classification results are ideal, as shown in red boxes 1, 2, 3, and 4, and most of the misclassified edge points of unripe and ripe fruit were corrected, as shown in red boxes 5 and 6.

Figure 9b shows the class change of the sample points in the enhanced classification compared to the preliminary classification. The red points denote no component types changed, and they were recorded as unchanged class points. The green points are the points that show that the component type changed, and they were recorded as changed class points. The points of the changed class are concentrated at the component edges, especially at the unripe and ripe fruit edges. In addition, there are more class change points at the leafstalk, whose diameter is smaller than the HSL footprint. Thus, inaccurate echo signals resulted in inaccurate spectral reflectance values. In addition, using a reprogramming strategy, the enhanced classification can correct edge misclassification points in persimmon tree samples, effectively improving classification accuracy.

**Figure 10:**
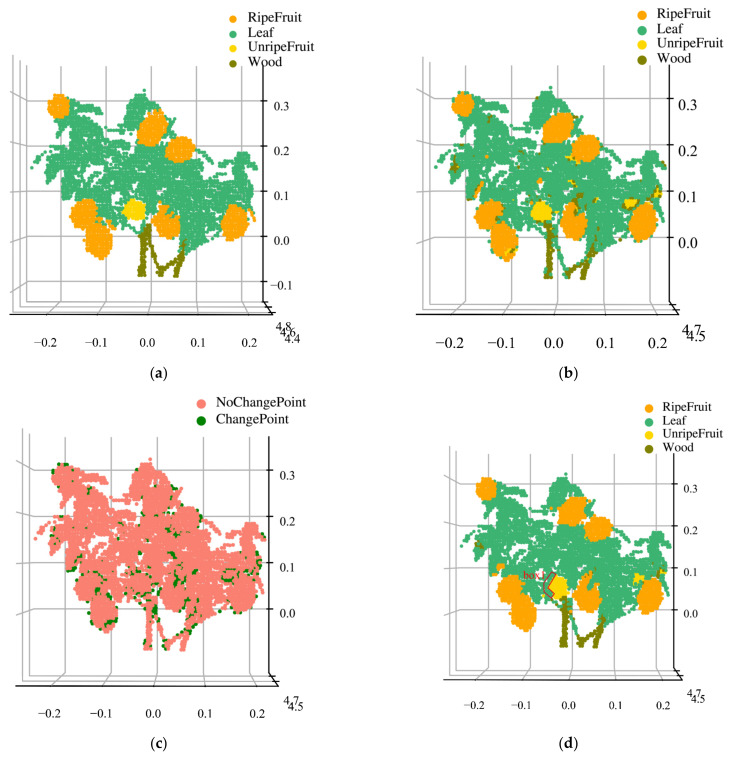
Reconstructed diagram of different algorithms for the classification of lemons. (**a**) Reconstruction of the real classification; (**b**) reconstruction of the preliminary classification; (**c**) class changes; and (**d**) reconstruction based on the proposed method results.

The classification results for the lemon components are shown in Figure 10. Figure 10a shows the true class. Figure 10b shows the reconstruction of the preliminary classification results; some misclassified points are at the edges of the ripe fruit. The class change points (Figure 10c) show that most of the corrected points are located in the edge area of each class. Four classes are distinguishable by their spatial–spectral features through our method, as shown in Figure 10d. Ripe fruit is clearly distinguished from leaves; however, unripe fruit edges are still slightly misclassified, such as in box 1. There are two reasons for the poor unripe component classification results: the low result of the preliminary classification of unripe fruit, while the result is used as an input for enhanced classification that will affect the accuracy; another is that the unripe fruit points are insufficient. In addition, chlorophyll contained in the shoots at the end of branches, which have similar reflectance curves to the leaves, is judged as a leaf in the preliminary classification, and the shoots were partially corrected with the enhanced classification.

## 5. Conclusions

We proposed a method for separating and classifying the wood, ripe fruit, unripe fruit, and leaves of persimmon trees with HSL measurements. Firstly, the spectral–spatial data of persimmon trees were acquired by HSL, and we classified each component of the samples via preliminary classification with spectral features. Then, based on understanding of edge point misclassification, we proposed an enhanced classification method to increase classification accuracy utilizing spatial information. Finally, we fused the classification results with 3D coordinates to visually reconstruct persimmon tree samples. The experimental results show that our method can be effectively used to classify the various components of fruit trees, providing a reference for further application. Additionally, our efforts will be directed towards integrating high-dimensional spectral and single-wavelength LiDAR spatial data to address differences in data structure and density between HSL’s spectral–spatial data and traditional 3D point cloud data. This will enhance the suitability of HSL data for 3D point cloud processing models.

Our future work aims to design a classification method that satisfies different fruit tree components in orchards and to develop a lightweight HSL system for more complex 3D modeling cases on agriculture and pomology.

## Figures and Tables

**Table 1:** Selected classification feature parameters.

Feature Parameters	Description
R_700_	Reflectance in the 700 nm band
R_730_	Reflectance in the 730 nm band
R_780_	Reflectance in the 780 nm band
R_850_	Reflectance in the 850 nm band
R_900_	Reflectance in the 900 nm band
AVG R_760_–R_930_	Average reflectance in the wavelength range from 760 nm to 930 nm
CI _red edge_	(R_780_/R_710_) – 1
NDVI	(R_800_ – R_670_)/(R_800_ + R_670_)
NDRE	(R_790_ – R_720_)/(R_790_ + R_720_)

**Table 2:** Comparison of the three classifiers’ accuracy.

Method	Accuracy (%)				
	Leaf	Ripe Fruit	Unripe Fruit	Wood	Overall
SVM	87.3	85.8	79.7	81.2	84.6
BPNN	96.7	80	86.2	67.8	86.3
RF	97.1	85.8	81.7	76.9	88.6

**Table 3:** Enhanced classification accuracy with different N values.

N/Number	9	10	11	12	13	14	15
Overall accuracy (%)	95.4	95.8	96.4	96.6	96.5	96.3	96.2

**Table 4:** Comparison of classification accuracy of the persimmon sample.

Method	Accuracy (%)				
	Leaf	Ripe Fruit	Unripe Fruit	Wood	Overall
Preliminary Classification	97.1	85.8	81.7	76.9	88.6
Enhanced Classification	99.4	98.2	94.6	89.1	96.6

**Table 5:** Comparison of classification accuracy of the lemon sample.

Methods		Accuracy (%)				
		Leaf	Ripe Fruit	Unripe Fruit	Wood	Overall
Preliminary Classification	SVM	87.2	64.5	56.1	69.9	80.1
	BPNN	86.5	78.5	88.4	78.8	84.9
	RF	89.3	83.3	82.3	75.5	88.3
Enhanced Classification		94.7	92.3	86	92.9	93.4

## Data Availability

Not applicable.
